# State-of-the-Art Cancer Biology, Biodiagnostics and Therapeutics in Japan

**DOI:** 10.3390/biomedicines11112905

**Published:** 2023-10-26

**Authors:** Junichi Yamaguchi, Eric di Luccio, Takaaki Hirotsu

**Affiliations:** Hirotsu Bioscience Inc., New Otani Garden Court 22F, 4-1 Kioi-cho, Chiyoda-ku, Tokyo 102-0094, Japan; e.diluccio@hbio.jp (E.d.L.);

Early cancer detection is key to improving patient survival and quality of life and reducing cancer treatments’ financial burden. The past several decades have seen the evolution of technologies for detecting signatures in cancer diagnosis, therapy, and prognosis. Although targeting multiple cancers based on common molecular mechanisms is convenient and cost-effective, the specialization of technologies to individual cancer types is indispensable due to differences in the developmental mechanisms of each cancer that exist in distinct organ with physically diverse characteristics. Operating from these two aspects and targeting single- and multi-cancer treatment, this Special Issue introduces current paradigms and recent advances or challenges in this field.

The first topic concerns the methods of multi-cancer screening and therapy. Although increasing the cancer screening rate affects early detection and reduces cancer-specific mortality, the rate remains low in the population, especially in Japan. New technologies for non-invasive cancer detection related to “liquid biopsy” have recently been developed with high sensitivity and reasonable cost-effectiveness. Some new technologies have already been launched into business and are available as a new service. The advent of such new products provides a glimmer of hope that we may improve the situation of the low screening rate. The review by di Luccio et al. focuses on the latest technology based on the scent detection of nematode (*C. elegans*), which can report the risk of at least 15 types of cancer from a small volume of urine samples taken from customers [1]. It describes not only the advantages of this method over legacy blood tumor markers CEA, CA19-9, and CA15-3, but also the success of discrimination of pancreatic cancer from other cancer types using mutant worms alongside normal ones. A service for risk testing pancreatic cancer is now also available in the market [1]. The two studies by Arao et al. and Tanaka et al. discuss molecular mechanisms of miRNA and a ubiquitously expressed gene Mint3, both of which are promising candidates for detecting and treating cancers and other diseases [2,3]. Existing miRNA variants with different modifications in terms of sequence substitutions, differences in length, and chemical modifications on specific nucleotides are found in the body fluid depending on whether the ailment relates to cancer, cardiovascular, neurodegenerative, or psychiatric disorders, or chronic inflammation. Transcriptome data from next-generation sequencing (NGS) help to construct personal profiles of divergent miRNAs, discriminating between each disease [3]. Currently, based on the technology of profiling miRNA variation, a commercially available cancer test has been launched, enabling discrimination between seven cancer types using urine samples available in the Japanese market. Regarding therapeutic targets for multi-cancer, Mint3 is one of the possible targets, belongs to the Munc-18-1 interacting protein (Mint) family, and is known as a non-essential gene in mice. The reduction of Mint3 is a compelling target for suppressing cancer aggressiveness since it can potentially increase cancer malignancy via inflammatory monocytes. Recently, one chemical compound, naphthofluorescein (Naph), was identified as its inhibitor. Evidence suggested that testing Naph in model mice of human breast and pancreatic cancer revealed the possibility for its application to clinical use. However, further fundamental studies are still required [2].

The second topic in this Special Issue highlights the recent advances in therapeutic and prognostic technologies, focusing in particular on individual cancer types. Different from multi-cancer detection methods, a consideration of the physical characteristics of each organ is often required for therapy and prognosis. In particular, pharmacotherapy in the central nervous system (CNS) remains challenging due to the blood–brain barrier (BBB). This is despite recognizing the importance of prognostic chemotherapy for CNS diseases and brain cancer to reducing the risk of recurrence. Tashima introduced current trends in Japan in drug delivery systems based on receptor-mediated transcytosis using monoclonal antibody conjugates [4]. To cross the BBB, drugs were attached to antibodies with high affinity for specific receptors such as transferrin or insulin receptors. Drugs can be transported into BBB together with the antibody–receptor complex via endocytosis; then, the drugs are released into the brain after exocytosis. As the success of the delivery itself is already well established, the challenge going forward is to increase the practical clinical use of these techniques in brain cancer treatment [4]. Related to the prognosis of brain cancer, treatment of radiotherapies for head and neck cancer requires that attention be paid to radiation-induced mucositis. This factor exerts a large influence on quality of life, especially in the mouth and throat. Next, Kawashita et al. revealed that the degree of radiation-induced mucositis can be predicted on the basis of patient lymphocyte counts at the pre-radiation stage [5]. Other studies focusing on individual cancer types are included in this Special Edition, with research on breast cancer [6], renal cell carcinoma [7,8], ovarian cancer from ovarian endometrioma [9], and inflammatory bowel disease (IBD), conditions which sometimes transform into cancer-prone phenotype [10]. Breast cancer is the most commonly diagnosed cancer in women, and Uchida et al. identified the nine candidate genes constituting therapeutic targets of breast cancer using existing databases. These were RNA-seq-based expression profiles taken from breast cancer tissues and CRISPR-Cas9/RNAi-based dependency maps of genes based upon the survival rate of cell lines derived from breast cancer tissue [6]. Clear cell renal cell carcinoma (ccRCC) is dominant in kidney cancer, the mortality rate for which has recently increased worldwide. Sabrina et al. demonstrated that the outcome is predictable by the status of the myeloid cells during immune checkpoint inhibition (ICI) therapy [7]. Kurota et al. showed the implication of the expression of the hemoglobin beta-chain in the tissue [8]. Ovarian cancer is often called the silent killer, being typically asymptomatic until an advanced stage. About half of ovarian cancer is related to ovarian endometrioma (OE), suggesting that in-depth observation of OE is effective for early detection. Kawahara et al. successfully improved diagnostic efficiency in discriminating endometriosis-associated ovarian cancer (EAOC) from OE using a novel index based on the patient’s age, tumor diameter, and R2 value in magnetic resonance relaxometry [9]. Moreover, Nakayama et al. discussed the current awareness of RNA modification in IBD [10]. Despite ample accumulated knowledge of related genes of IBD, our comprehension of the genetic changes responsible for this disease’s pathogenesis are still limited. Gene regulation via the chemical modification of RNA may play an essential role in the pathogenesis of IBD. In recent years, with the advent of NGS, an innovative approach to “genomic medicine” has emerged that focuses on the responsible genomic changes for the pathogenesis of cancer in each patient. Although the cancer subtype used to be defined according to organs, it is now changing to take into account the individual differences of each patient. Thus, treatment will be performed via optimization to the personal genomic profile. In Japan, since 2019, the practical use of this approach has been started under the national healthcare system. This approach is a potential biotechnological breakthrough, and its use will inevitably increase in the future, although recently only 10–20% of such studies have led to the development of actual treatments. Considering the increasing number of cancer patients, attempts to further advances in conventional knowledge and advance the methods discussed in this Issue remain as crucial as proceeding with upcoming innovative approaches.
